# An area efficient and high throughput implementation of layered min-sum iterative construction a posteriori probability LDPC decoder

**DOI:** 10.1371/journal.pone.0249269

**Published:** 2021-03-29

**Authors:** Hasnain Raza, Syed Azhar Ali Zaidi, Aamir Rashid, Shafiq Haider

**Affiliations:** Electronics Engineering Department, Faculty of Electronics and Electrical Engineering, University of Engineering and Technology, Taxila, Pakistan; Effat University, SAUDI ARABIA

## Abstract

Area efficient and high speed forward error correcting codes decoder are the demand of many high speed next generation communication standards. This paper explores a low complexity decoding algorithm of low density parity check codes, called the min-sum iterative construction a posteriori probability (MS-IC-APP), for this purpose. We performed the error performance analysis of MS-IC-APP for a (648,1296) regular QC-LDPC code and proposed an area and throughput optimized hardware implementation of MS-IC-APP. We proposed to use the layered scheduling of MS-IC-APP and performed other optimizations at architecture level to reduce the area and to increase the throughput of the decoder. Synthesis results show 6.95 times less area and 4 times high throughput as compared to the standard min-sum decoder. The area and throughput are also comparable to the improved variants of hard-decision bit-flipping (BF) decoders, whereas, the simulation results show a coding gain of 2.5 over the best implementation of BF decoder in terms of error performance.

## 1. Introduction

Low density parity check (LDPC) codes [1,2] are used in many communication systems [3] and are also of particular interest in data storage systems [4] due to their excellent error correction capability. With the increasing demand of high data rates in next generation communication systems, it is required to implement a very high speed decoder that should also meet the area and power requirements of the communication standard. Because of the inherent parallelism in their encoding and decoding algorithms, it is possible to implement a very high throughput encoder and decoder of LDPC codes. The low complexity variants of the soft-decision iterative message passing belief propagation (BP) algorithm, such as min-sum (MS) [5], offset min-sum [6] and scaled min-sum [7], are usually the choice of hardware implementation because of their excellent error correction performance close to the Shannon limit. Two types of scheduling are used in all these message passing algorithms namely, flooding schedule [8] and layered scheduling [9] depending upon how messages are propagated between the check nodes and variable nodes of the parity check matrix. In the flooding schedule, the check nodes and the variable nodes compute their messages subsequently in each iteration. Whereas, in layered scheduling the rows of parity check matrix is divided into layers and the messages passed by the check nodes are immediately used to update the log likelihood ratios (LLRs) variable nodes within the processing of one layer. Therefore, the processing of next sequential layer uses the update values of LLRs. The number of iterations are reduced to half in layered scheduling as compared to flooding schedule which helps in the implementation of high speed decoders.

Many prior works have implemented high speed LDPC decoders based on the low complexity variants of BP algorithm. The main challenge in these implementations is the selection of parallelism in order to meet the area and throughput requirements of the communication standard, design of the routing network, the placement of data in memories in order to avoid the memory conflicts during the read and write of check node and variable node messages, and dealing with the pipeline hazards in the pipelined layered architecture of LDPC codes. In [10], a block level parallel architecture for quasi-cyclic (QC) LDPC codes is implemented, where, all the rows in a layer are processed in parallel. The variable node and check node processors are optimized and a flexible routing network is used between the LLR memory and the processors in order to adapt the architecture to various parity check matrices. The routing network is implemented with the help of barrel shifters, where, the shift factors are stored in a memory. The authors in [11] have implemented pipelined layered decoder architecture for QC-LDPC codes. A high throughput is achieved by implementing a flexible partially parallel decoder supporting different parallelism factors and a routing network supporting different matrices. Single port memory banks are used and an offline algorithm is used to organize the messages in these memory banks in order to avoid memory access conflicts for processing large number of rows in parallel. Similarly the offline algorithm is used to relax the messages read access constraints in order to avoid the read after write hazard in the pipelined architecture. Many works have implemented a fully parallel and unrolled LDPC decoder architecture [12–14]. In a fully parallel unrolled decoder architecture is implemented with a throughput of 588 Gbps for high speed optical and Ethernet networks. However, these fully parallel and ultra high throughput decoders are implemented at the expense of large area.

Another class of decoding algorithms called the hard-decision algorithms result in very low complexity decoders but at the cost of reduced error correction performance. Among the hard-decision algorithms are the bit-flipping (BF) algorithms and the majority-logic decoding algorithm. Many researchers have proposed changes in these hard-decision algorithms in order to improve the error correction performance and to maintain a reasonable hardware complexity [15–17]. However, the error correction performance of these algorithms is still low as compared to the MS algorithm, especially, at low frame error rate.

In this paper, we have analyzed another class of decoding algorithm called the Gradient-Projection (GP) decoding of LDPC codes proposed by Kasparis and Evans in [18]. The GP decoding algorithm is based on formulating a non-linear multimodal objective function, which include all the parity check constraints, and then finding the global minimum of this objective function by using the gradient projection method. The authors also proposed the variations of GP decoding algorithm in [19], called the Iterative Construction of A Posteriori Probability (IC-APP) and MS-IC-APP (min-sum variant) decoder, which results in reducing the complexity of the GP decoder and at the same time linked the GP algorithm with the BP algorithm and its low complexity variants. The authors showed that the GP algorithm and its variants perform close to the MS algorithm, especially, for geometry-based LDPC codes. In this paper, we have used the MS-IC-APP algorithm for the decoder implementation. We analyzed the performance of MS-IC-APP for a regular quasi-cyclic (QC) LDPC code and compared the performance with MS algorithm and the improved variants of the hard-decision BF algorithms. We implemented an area optimized and high throughput hardware of the MS-IC-APP decoder. In this regard, we also proposed to use the layered version of the MS-IC-APP algorithm (similar to the layered MS decoding proposed in [9]), which results in the elimination of the check-to-variable (CTV) message memory, and therefore, results in large area savings. The permutation unit in our proposed implementation is optimized by replacing the large barrel shifters with multiplexers at the input of a single check node processor. This results in further reducing the area and shortening the critical path, thus, increasing the frequency and the throughput of the decoder. Simulation and implementation results show better error correction performance of the layered MS-IC-APP algorithm, especially, at low frame error rate and comparable hardware complexity as compared to the hard-decision BF algorithms.

The rest of the paper is organized as follows: Section 2 gives the introduction about the LDPC codes and presents the algorithm of layered MS-IC-APP. Section 3 gives the simulation results of the layered MS-IC-APP algorithm for a (648,1296) regular QC-LDPC code. The proposed hardware architecture of the layered MS-IC-APP decoder is discussed in Section 4. Section 5 gives the synthesis and simulation results of the layered MS-IC-APP algorithm for (648, 1296) regular QC-LDPC code and shows the comparison of the results with the state of the art implementations. Section 6 concludes the paper.

## 2. Background

### 2.1. LDPC codes

A binary LDPC code is described by a sparse parity check matrix, **H**, having dimension *M*×*N*, where, *N*>*M*. A valid codeword $\hat{x}$ of *N* bits should satisfy $H∙{\hat{x}}^{T}$, where ${\hat{x}}^{T}$, denotes the transpose of $\hat{x}$. The codeword *r* received from the channel could have an error whose probability depends upon the underlying communication channel. E.g. for a binary symmetric channel (BSC), the crossover probability, *β*, shows the number of bits that are likely to be flipped in the transmitted code-word $\hat{x}$. In this paper, we have used the BSC for performance evaluation and decoder implementation. The number of 1’s in a row and column of **H** is called the row and column degree, respectively. A regular parity check matrix has equal number of 1’s in all the rows/columns, whereas, an irregular matrix has variable degree across different rows/columns. The parity check matrix is also categorized as structured or unstructured depending upon whether it has a regular structure or not. The QC-LDPC codes are a class of structured LDPC codes, where, the parity check matrix consists of *M*_*b*_×*N*_*b*_ array of *Z*×*Z* circulant permutation sub-matrices. The number of 1’s in a row or a column of a circulant sub-matrix, *θ*_*i*,*j*_, is the weight of the sub-matrix. The weight *w* of the sub-matrix can be 0, 1 or higher and therefore, the sub-matrix can be either a zero matrix, cyclically shifted identity matrix or multiple independent cyclically shifted identity matrices superimposed in a sub-matrix, respectively. The regular structure of LDPC codes result in a simplified architecture of the encoder and decoder. The parity check matrix is represented graphically with the help of a bi-partite Tanner graph. The Tanner graph consists of *N* variable nodes (VNs) (which correspond to the columns of **H**) and *M* check nodes (CNs) (which correspond to the rows of **H**), where, the connection between a VN and CN denotes that the entry in the corresponding row and column of **H** is equal to 1.

### 2.2. MS-IC-APP algorithm

As mentioned in the previous section, we have used the low complexity min-sum variant of the IC-APP algorithm, proposed in [‎19], called the MS-IC-APP algorithm. Similar to BP, messages are exchanged between VNs and CNs in MS-IC-APP for gradually updating the reliability values of the VNs towards correct values. The horizontal layered scheduling of the MS-IC-APP algorithm is described in Algorithm 1. The following notations are used in this algorithm:

- $L_{j}$ is the a-priori log-likelihood ratio (LLR) computed from the bits *r*_*t*_ received from the channel given as log $\left(\frac{P_{r}(x_{t}=0|r_{t})}{P_{r}(x_{t}=1|r_{t})}\right)$
- $\gamma_{ij}^{\left(k\right)}$ is the CTV message given by the *ith* CN to the *jth* VN in the *kth* iteration.
- *N*(*i*) is the set of VNs connected to the *ith* CN and *N*(*i*)\*j* is the set of VNs connected to the *ith* CN except VN *j*.
- $r_{ij}^{\left(k\right)}$ and $R_{j}^{\left(k\right)}$ denote the variable to check (VTC) message from the VN *j* to CN *i* and the a posteriori LLR of VN *j* at *kth* iteration, respectively.

**Algorithm 1:** Layered MS-IC-APP decoding algorithm.

**1. Initialization**:

∀ *VN*_*j*_, *j*∈{1,…,*N*} do $R_{j}^{\left(0\right)}=L_{j}$

**2. Check node processing**:

∀ *CN*_*i*_, *i*∈{1,…,*M*} and ∀ *VN*_*j*_, *j*∈{1,…,*N*} do.

$$r_{ij}^{\left(k\right)}=R_{j}^{\left(k-1\right)}$$ (1)

$$\gamma_{ij}^{\left(k\right)}=\prod_{j^{\prime }\in N(i)\backslash j}\mathrm{sgn}\left\{r_{ij^{\prime }}^{k}\right\}\times \underset{j^{\prime }\in N(i)\backslash j}{\min}\left|r_{ij^{\prime }}^{\left(k\right)}\right|\times \alpha$$ (2)

$$R_{j}^{\left(k\right)}=r_{ij}^{\left(k\right)}+\gamma_{ij}^{\left(k\right)}$$ (3)

Estimated code-word is $\hat{X}=({\hat{x}}_{1},{\hat{x}}_{2},\ldots ,{\hat{x}}_{N})$, where element ${\hat{X}}_{j}$ is calculated as:

$${\hat{x}}_{j}=\left\{ \begin{array}{l} 0\;if\;R_{j}^{\left(k\right)}\geq 0\\ 1\;else \end{array}\right.$$ (4)

If $H{\hat{x}}^{T}$ = 0 then stop, with correct code-word $\hat{x}$, otherwise go to the step 2.

From Algorithm 1, we can see that in each iteration k, the LLRs are directly given to the CNs as VTC messages as compared to the standard min-sum algorithm in which the VTC message is computed as: $r^{\left(k\right)}=R_{j}^{\left(k\right)}-\gamma_{ij}^{\left(k-1\right)}$ This modification in the MS-IC-APP algorithm results in reduction of hardware as compared to the MS algorithm but at the cost of reduced error correction performance. In order to further reduce the area, we have used the layered version of the MS-IC-APP algorithm in this work instead of flooding schedule. In the layered algorithm, the *M* rows of **H** are divided into *L* different layers, where, each layer consists of *N*_*L*_ = *M*/*L* rows. The layers are processed sequentially and all the rows in a layer are processed in parallel. During the processing of a layer, all the computed CTV messages are used to update the LLRs of the corresponding VNs, as given in Eq 3, and therefore, the next sequential layer uses the updated LLRs of VNs. The modification of VTC message and the layered scheduling results in the elimination of CTV message memory in MS-IC-APP and therefore, results in large reduction of the area of decoder. The number of iterations in the layered algorithm reduces to half and therefore, results in doubling the throughput of the decoder as compared to the flooding schedule. In this work, we have implemented the LDPC decoder hardware of a (3,6) regular QC-LDPC code described by a base matrix of size *M*_*b*_×*N*_*b*_ = 12×24, and expansion factor *Z* = 54, relating to a parity check matrix of dimension *M*×*N* = 648×1296.

**Fig 1:**
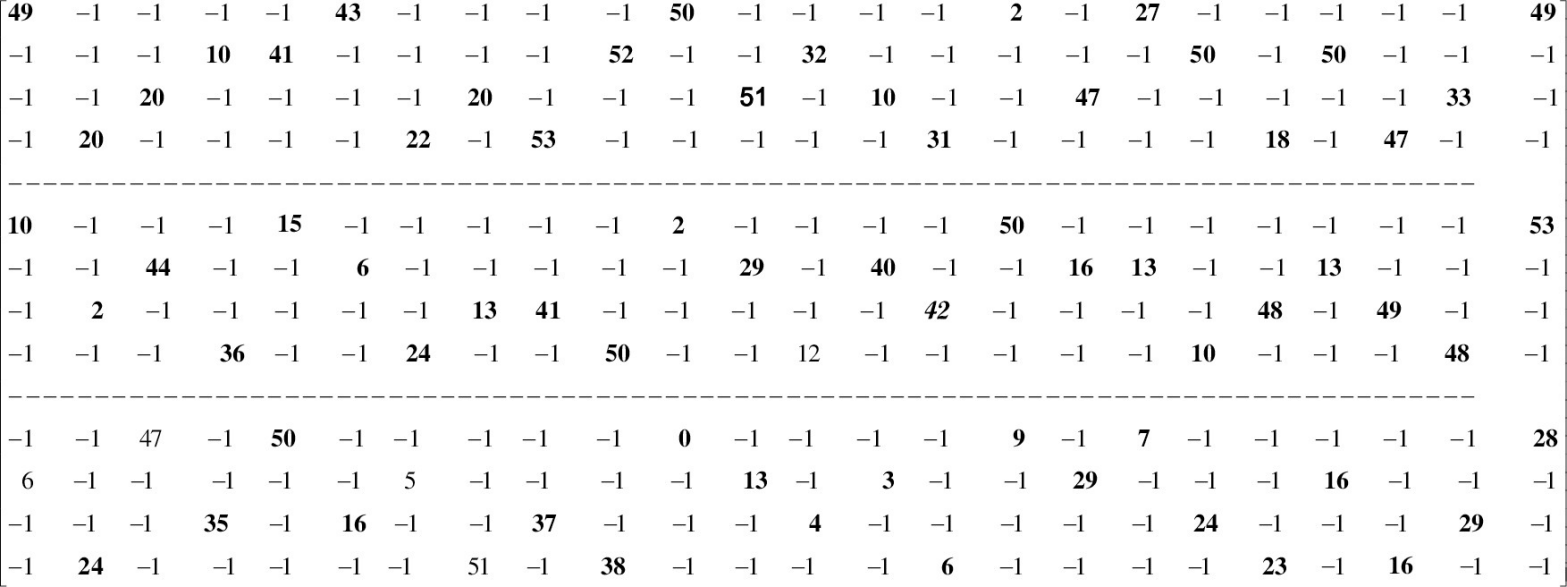
Base matrix of (648,1296) regular QC-LDPC code with a column and row degree of 3 and 6, respectively.

The base matrix is shown in Fig 1. The base matrix is divided into three layers, where, each layer consists of 4 rows of circulants i.e. 4×54 = 216 rows of **H**. There are six cyclically shifted identity sub-matrices, represented by non-negative entries, in each row of the base matrix, where, the non-negative number shows the corresponding shift factor. We denote these sub-matrices with *C*_*st*__*L*_*p*_ where, *s* = 1:4 shows the corresponding row of circulants in a layer *p*, and *t* = 1:6 shows the corresponding non-negative column of circulant in a row *s*.

## 3. Layered MS-IC-APP error-correction performance

We performed the monte carlo simulations for analyzing the error correction performance of (648,1296) QC-LDPC code at different number of quantization bits of LLRs. For this purpose, a simulator of the whole communication system with QC-LDPC code encoder, decoder and the BSC is implemented in C. For the binary symmetric channel, a 32-bit linear feedback shift register (LFSR) is implemented and the random values produced by the LFSR are divided by the maximum value of LFSR i.e. 2^32^ and then the result is compared with the value of BSC crossover probability *β*. These noise bits with the probability of 1 as *β* is XORed with the bits sent from the transmitter.

**Fig 2:**
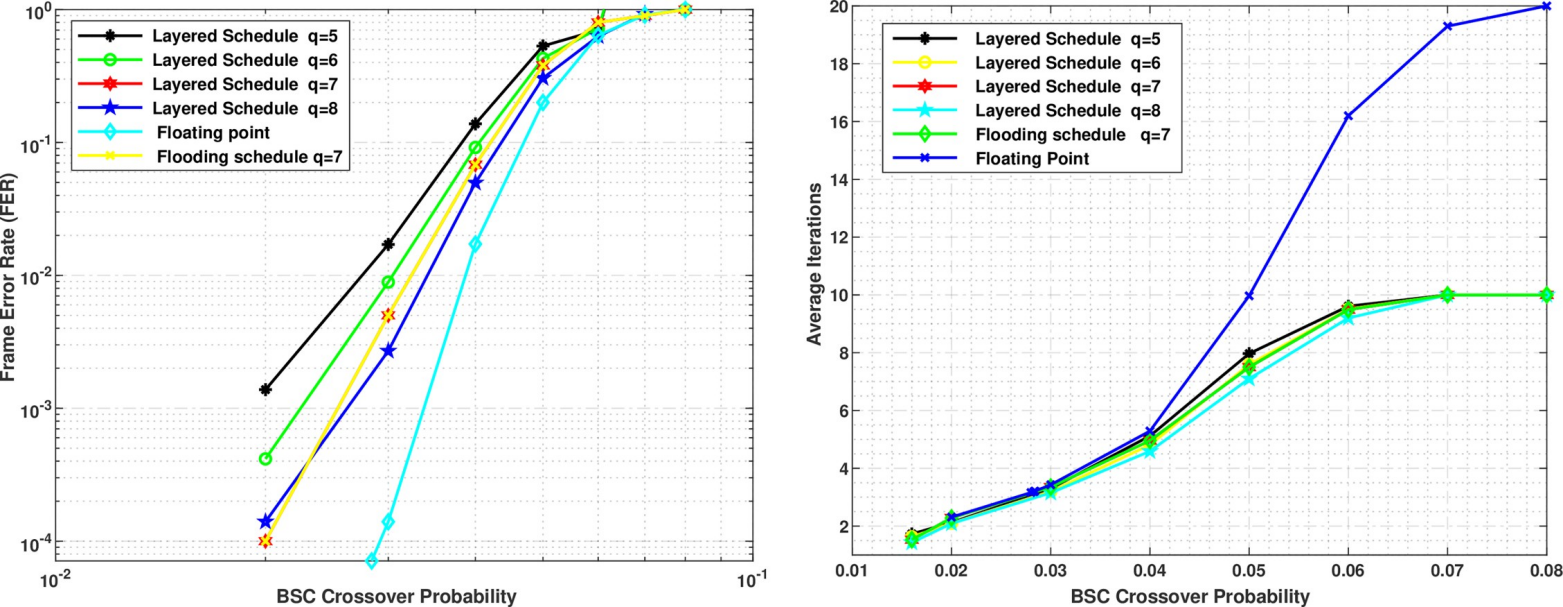
Simulation results of MS-IC-APP for (648,1296) QC-LDPC code with different number of quantization bits of LLRs. (a) Frame error rate (b) Average numbers of Iterations.

The parameters for the simulations are set as follows: We used *q* bits for $L_{j}$ and *Q* = *q*+1 bits for *R*_*j*_. We measured the value of frame error rate (FER) at different values of *β*, where, a total of 1×10^7^ frames are simulated for each value of *β* and the simulations are stopped when 100 wrong frames are measured at a particular value of *β*. The maximum number of iterations is set as 20. The scaling factor alpha ∝ = 0.5 is used. Fig 2 shows the simulation results of (648,1296) QC-LDPC code using different values of *q* in layered MS-IC-APP. The simulation results for the floating point implementation and the flooding schedule of MS-IC-APP for q = 7 are also given for comparison purposes. As the number of rows in the chosen layers of the base matrix to be processed in parallel does not contain two 1’s in a single column, therefore, there is no degradation in the performance of layered schedule as compared to the flooding schedule. This is evident from the figure for the case of *q* = 7. From the figure we can see that the performance increases slightly with the increases in number of quantization bits. E.g. there is a difference of *β* = 0.005 approximately at a FER of 1×10^−3^ between *q* = 5 and *q* = 7. The figure also shows that the results of *q* = 7 and *q* = 8 are very close to each other.

## 4. Hardware architecture of layered MS-IC-APP algorithm

**Fig 3:**
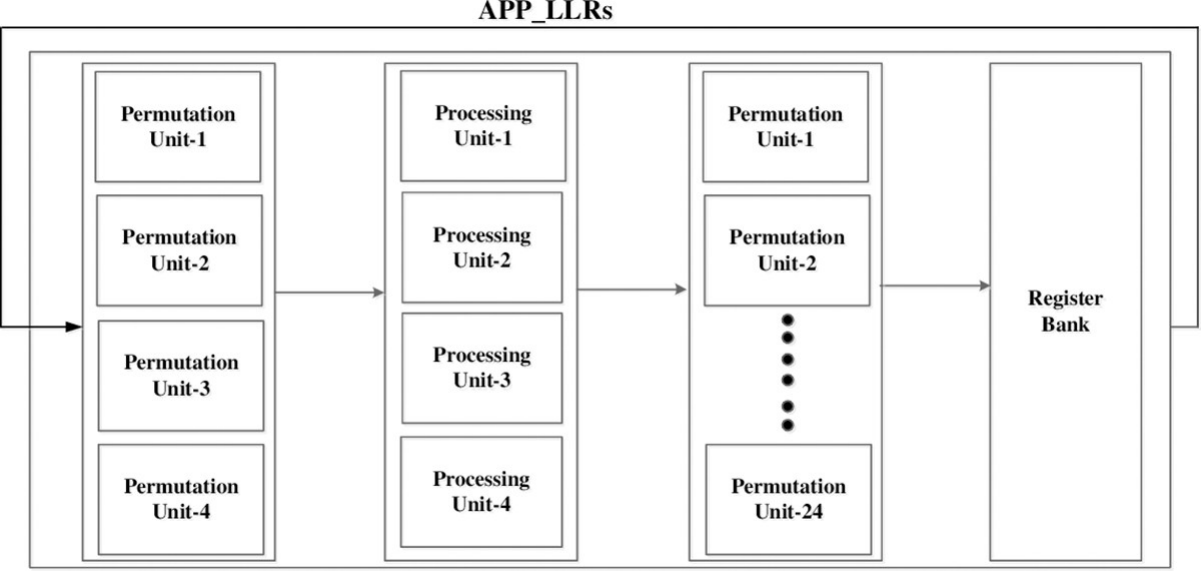
Top level block diagram of the decoder.

The overall block diagram of the proposed MS-IC-APP decoder hardware for (648,1296) regular QC-LDPC code is shown in Fig 3. The register bank is used to store the a-priori LLRs received from the channel and the updated a-posteriori LLRs received from processing units in each iteration. The register bank consists of 24 registers, where, each register is an array of flip-flops for storing the corresponding 54 LLRs of 1 column of circulants. There are four Processing Units in the hardware which are responsible for processing all 216 rows of a layer, where, each processing unit process 1 row of circulants, i.e. 54 rows, in parallel in a layer. The processing units receive LLRs from the register bank through the permutation units. Similarly, the updated LLRs from the processing units are given to the register bank through permutation units.

**Fig 4:**
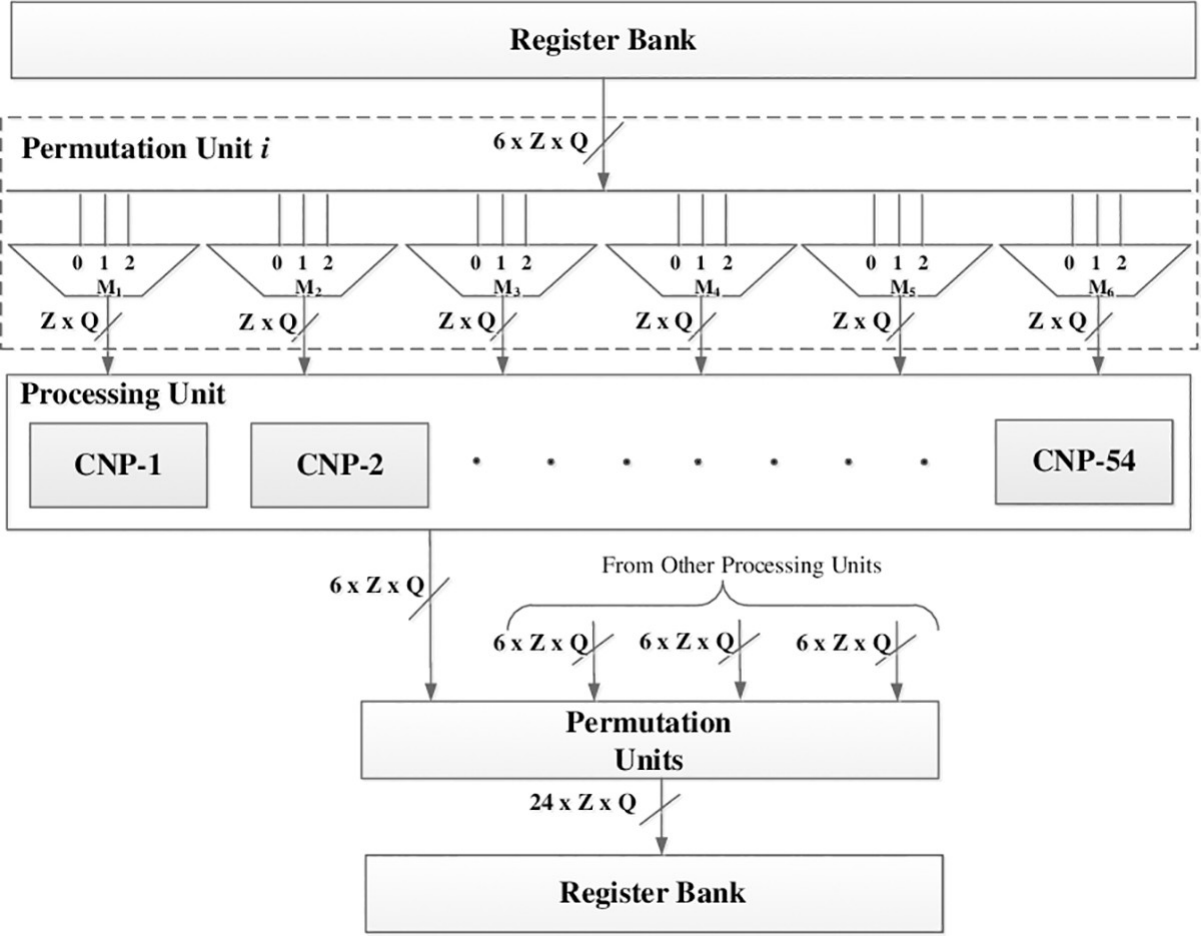
Detailed data flow diagram for processing 1 row of circulants in a layer.

The permutation units at the input of processing units consists of blocks of MUXes for selecting the required LLRs corresponding to *C*_*st*__*L*_*p*_ for all processing units, and therefore, total 6 × 54 LLRs are given to 1 processing unit for processing 1 row of circulants in layer *p*. These permutation units also rotate the LLRs corresponding to the shift factor of *C*_*st*__*L*_*p*_. E.g. for *C*_11__*L*_0_ = 49, a left rotation of 49 is performed on the first set of Z LLRs given to the first processing unit in layer 0. Fig 4 shows the case of processing 1 row of circulants in a layer. In this figure, a single permutation unit is shown at the output of register bank. There are 6 blocks of MUXes in a permutation unit *i* and each block consists of *Z* = 54 MUXes. These blocks of MUXes select and rotate 6 set of *Z* LLRs for the processing unit corresponding to the processed layer *p*, where, *p* = 0 to 2. Similarly, there are 24 permutation units at the input of the register bank, where, each permutation unit consists of a block of 54 MUXes for rotating and selecting the correct updated LLRs for each register from the processing units.

**Fig 5:**
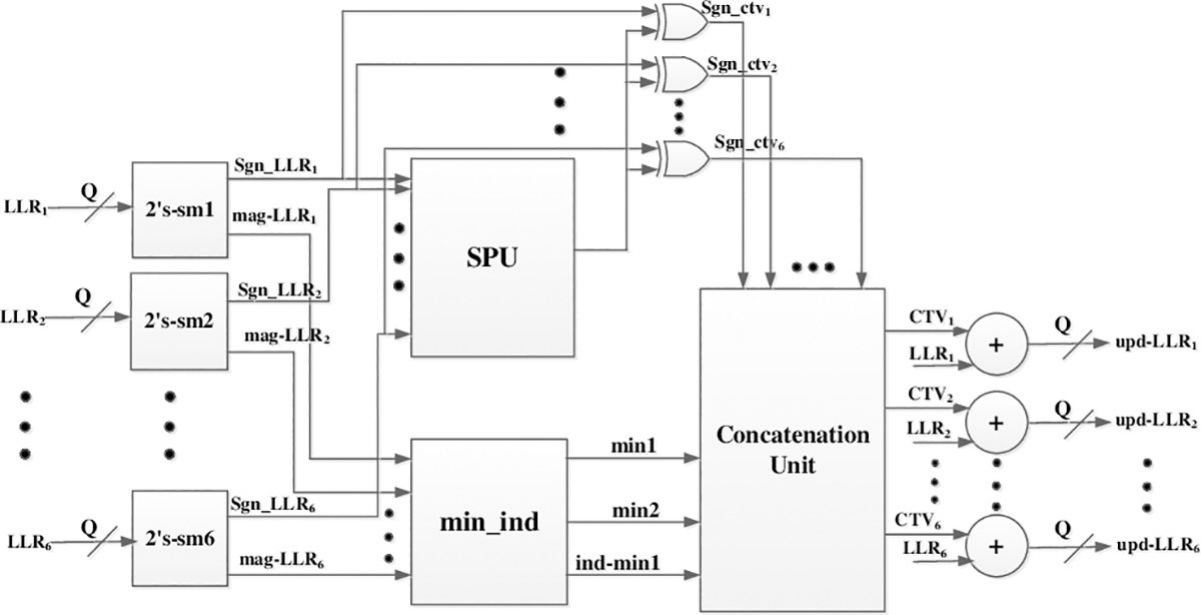
Architecture of a Check Node Processor (CNP).

The processing unit consists of 54 check node processors (CNPs) which process the whole row of circulants i.e. 54 rows in parallel. Each CNP receives 6 LLRs from the permutation unit as VTC messages, compute the CTV messages and output the updated value of these LLRs by adding the CTV messages with the corresponding LLRs. The internal architecture of CNP is shown in Fig 5. There are 6 units in CNP for converting the corresponding LLRs of VNs into sign-magnitude format. The SPU block is then used to find the product of all the signs of LLRs and the *min*_*ind* block is used to find the first two minimums and the index of the first minimum from the absolute values of LLRs. The tree like architecture for finding the first two minimums and index of first minimum as proposed in [20] is used for the *min*_*ind* block. The XOR gates are used to exclude the sign of each LLR from the sign product in order to compute the final sign of each CTV message, denoted as *sgn*_*ctv*_*i*_, where, *i* = 1:6. The concatenation block receives all the signs of CTV messages, the first two minimums and the index of the first minimum. The minimum values are then scaled by the scaling factor ∝. The concatenation block computes the magnitude of all CTV messages by comparing the index of first minimum with the index of each LLR and gives 2nd minimum as magnitude of CTV message for the LLR whose index is equal to the index of the first minimum, whereas, the first minimum is given to the rest. The concatenation unit gives CTV messages as output (denoted as CTV1 to CTV6 in Fig 5) by combining the sign of each CTV message with the magnitude and converting it to the 2’s compliment format. The CTV messages are then added to the corresponding LLRs and quantized to *Q* bits. These updated LLRs from all CNPs are then given to the permutation unit for rotating and then storing in the correct register in the register bank. As mentioned in the previous section, we have employed layered decoding of MS-IC-APP algorithm. Due to the layered decoding, all the CTV messages are used in the current iteration for updating the LLRs and are not required in the subsequent iteration. Therefore, the CTV message memory is eliminated in the proposed hardware which results in large area saving of the decoder.

## 5 Hardware implementation and simulation results

The architecture of layered MS-IC-APP decoder, as described in the previous section, is implemented using Verilog HDL. We used 7 bits for the intrinsic information from the channel i.e. a priori LLRs and 8 bits for the a-posteriori LLRs. The bits are chosen based on the simulation results given in section 3. The Verilog Model is synthesized by targeting the 90 nm CMOS standard cell library and using Leonardo Spectrum tool from Mentor Graphics. The synthesized area, maximum achieved frequency of the decoder after synthesis and the throughput at a particular FER is shown in Table 1. The throughput of the hardware is calculated based on the following formula:

$${TP}_{dec}=\frac{f_{max}\times N}{N_{avg\_iters}\times N_{clk/iters}}$$ (5)

where, *N*_avg_iter_ is the average number of iterations for decoding 1 frame and *N*_*clk*/*iter*_ is the number of clocks for completing 1 iteration of the decoder.

**Table 1:** Area and throughput comparison with state of the art implementations for (648, 1296) QC-LDPC code.

	Area (mm^2^) (technology)	Area (mm^2^) (scale d to 90nm)	f_max_ (MHz)	N_clk/iter_	TP(Gbps) @ 1×10^−5^ FER	TAR Gbps/mm^2^
**GDBF [‎17]**	0.088 (65nm)	0.17	160.28	1	103.97 (Navg_iter = 2.00)	611.59
LSFR-PGDBF S = 4Z [‎17]*	0.10 (65nm)	0.19	168.95	1	62.17 (Navg_iter = 3.50)	327.21
IVRG-PGDBF S = 4Z [‎17]*	0.093 (65nm)	0.18	168.95	1	62.17 (Navg_iter = 3.50)	345.39
**MS [‎10]**	0.72 (65nm)	1.38	180.56	6	16.66 (Navg_iter = 2.34)	12.07
VNSA-PGDBF *p*0 = 0.7 [‎23]**	0.32 (90nm)	0.32	370	1	99.3 (Navg_iter = 4.83)	310.31
VNSA-IM-PGDBF *p*0 = 0.7 [‎23]**	0.29 (90nm)	0.29	400	1	81.3 (Navg_iter = 6.38)	280.34
Layered MS [‎22]***	0.85 (40nm)	4.30	249.78	-	1.69–5.067	0.39–1.18
**This work**	0.188 (90nm)	0.188	220	3	68.37 (Navg_iter = 1.39)	363.67

*S is register size of random sequence generator and its size, taken as integer multiples of circulant size Z, effect the hardware complexity and the performance of PGDBF.

**p0 and (1-*p*0) are the functions of type-1 and type-2 VNUs in VNSA through which the random sequence generator function of PGDBF is implemented in VNSA, therefore, removing the need of random sequence generator.

***The hardware implementation supports three different frame lengths, FL = 648, 1296 and 1944, used in the IEEE 802.11 n/ac/ax.

For comparison purpose, we have also given the results of the hardware implementation of the same QC-LDPC code from the prior state of the art works in Table 1, where, both the MS and the improved variations of the BF algorithm are included. The results of the previous hardware implementations are scaled to 90 nm technology for fair comparison. The area and frequency are scaled by the factor 1/K^2^ and K, respectively, where K is the ratio of two different technologies [21]. From the table we can see that the area efficiency of the proposed hardware is very high as compared to the layered MS implementation in [10] and [22]. whereas, the area of the proposed decoder is also comparable to the improved variants of the hard-decision BF algorithm. Due to a high achieved frequency and reduced average iterations of MS-IC-APP algorithm, the throughput measured at a FER of 1×10^−5^ is 68.37 Gbps which is 4 times as compared to the MS decoder in [10]. The throughput is also high as compared to the PGDBF algorithm, which is the best available implementation of the improved BF algorithm in terms of error performance. The authors in [23] have proposed a variable-node-shift architecture (VNSA) based approach for implementing the PGDBF algorithm. Their implementation results in a high throughput hardware, however, with an increase in the area as compared to the implementations in [17]. We have also given the throughput to area ratio (TAR) in the last column of Table 1. Apart from GDBF algorithm, the proposed implementation has the highest TAR as compared to the PGDBF and MS implementations. These results show the high area-efficiency and throughput performance of the proposed implementation.

**Fig 6:**
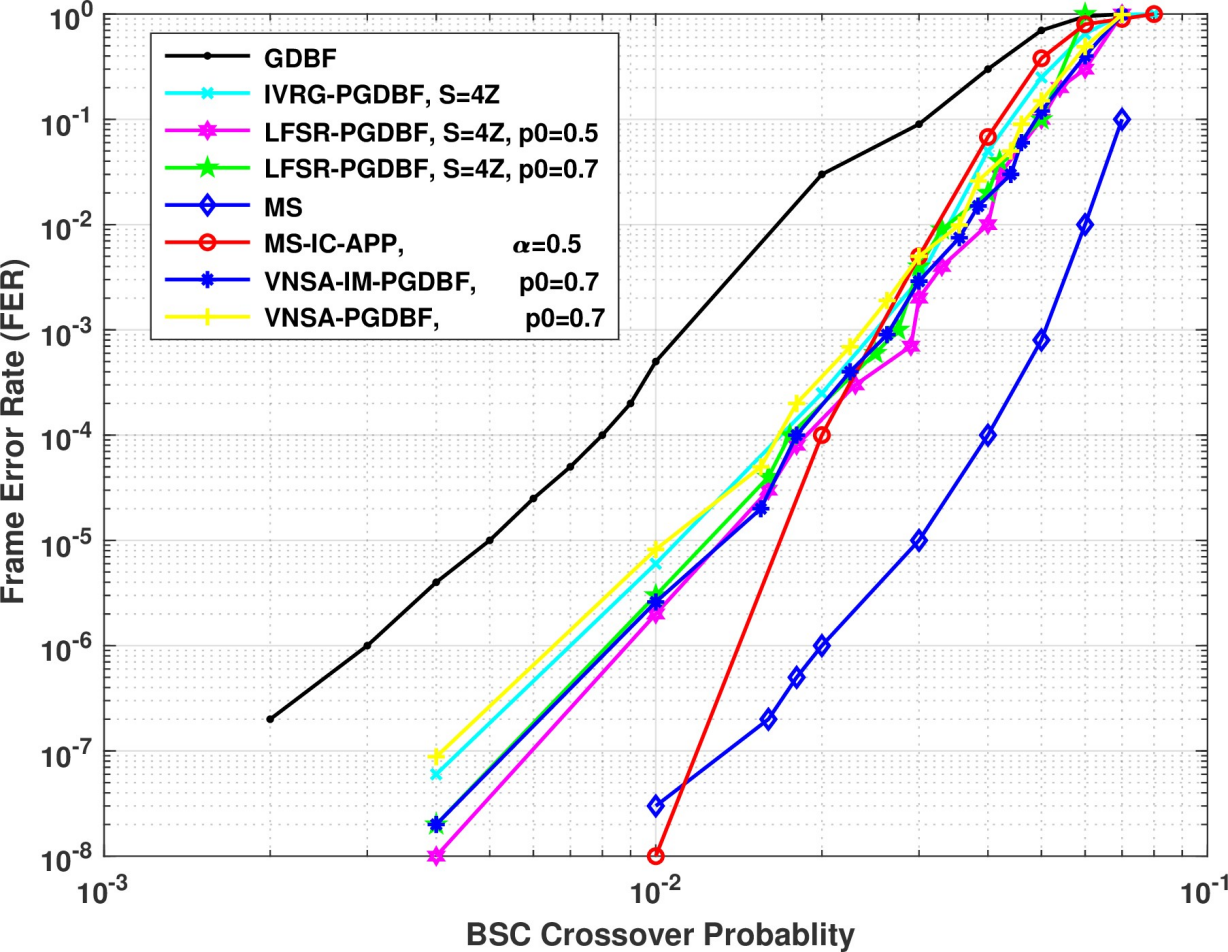
Comparison of error correction performance of layered MS-IC-APP algorithm with state of the art implementations for (648,1296) QC-LDPC code.

For comparing the error performance of the layered MS-IC-APP algorithm, we have given the FER results of the proposed and state of the art implementations in Fig 6. The result of the proposed decoder is given for *q* = 7 bits and a scaling factor ∝ = 0.5. From the figure we can see that the layered MS implementation has the best error correction performance, whereas, the GDBF performance is low than all other algorithms. From the figure we can see that the layered MS implementation has the best error correction performance, whereas, the GDBF performance is low than all other algorithms. The MS-IC-APP algorithm error performance is close to the LFSR-PGDBF algorithm till FER of 1×10^−4^, whereas, at low FERs the performance of the MS-IC-APP algorithm is better than the LFSR-PGDBF and IVRG-PGDBF. E.g. there is a difference of *β* = 0.006 at a FER of 1×10^−8^ between MS-IC-APP and LFSR-PGDBF with *S* = 4*Z* and *p*0 = 0.7 (please refer to [‎‎17, ‎23] for details of S and *p*0, respectively) and hence shows a coding gain of 2.5 as compared to the LFSR-PGDBF. The improvement in the performance is due to the fact that the MS-IC-APP algorithm works on the soft-decision LLRs from the channel instead of hard-decision bits from the receiver for BSC in case of GDBF, PGDBF and VNSA-PGDBF. The MS-IC-APP algorithm performance is low as compared to the MS algorithm as reasoned in section 2.2. However, at low values of cross-over probability, the average number of iterations for the MS-IC-APP is very low (as given in Table 1, average iterations is 1.39 at a FER of 1×10^−5^) and therefore, the performance becomes close to MS algorithm as shown in the figure. The performance is even better for the cross-over probability of 0.01 which shows the good error correction performance of MS-IC-APP.

## 6. Conclusion

In this work, we analyzed the low complexity variant of a different class of LDPC code decoding algorithm called the MS-IC-APP algorithm. We proposed an area-efficient and high throughput hardware implementation of the MS-IC-APP algorithm. We used the lay-ered scheduling of the MS-IC-APP in order to eliminate the CTV message memory and also performed some other optimizations in the hardware in order to optimize the area-efficiency of the decoder. The layered scheduling also results in increasing the throughput of the decoder. We presented the synthesis and simulation results of the proposed decoder for a (648,1296) regular QC-LDPC code and compared the results with the state of the art implementations of hard-decision BF algorithms and the standard MS algorithm for the same code. Results show that the proposed implementation has 6.5 times less area and 4 times high throughput as compared to the layered MS implementation, whereas, the area and throughput is comparable to the LFSR-PGDBF implementation. The simulation results show that the layered MS-IC-APP achieves a coding gain of 2.5 at a FER of 1×10^−8^ over the LFSR-PGDBF.

## Supporting information

S1 Algorithm MATLAB code to generating the (648,1296) matrix by using QC-LDPC matrix. (DOCX)
