# Supplementary material for: An area efficient and high throughput implementation of layered min-sum iterative construction a posteriori probability LDPC decoder
Source: PLoS One. 2021 Mar 29;16(3):e0249269. doi: 10.1371/journal.pone.0249269 (PMC8007033; doi:10.1371/journal.pone.0249269)
Supplement: S1 Algorithm — (DOCX) [file pone.0249269.s001.docx]

**S1 Algorithm**

**MATLAB code to generating the (648,1296) matrix by using QC-LDPC matrix.**

clear all

Hb=load(“QC_mtarix”);

cs = 54;

Z = false(cs,cs);

I = false(1,cs);

%I(1) = 1;

[Ny Nx] = size(Hb)

for i= 1:Ny

for j = 1:Nx

if (Hb(i,j) == -1)

H((i-1)*cs+1:i*cs,(j-1)*cs+1:j*cs) = Z;

else

I(Hb(i,j)+1) = 1;

for k=1:cs

IS(k,:) = I;

I = [I(end) I(1:end-1)];

end

H((i-1)*cs+1:i*cs,(j-1)*cs+1:j*cs) = IS;

end

I = false(1,cs);

end

end
